# Harnessing functional feed additives for sustainable production: the role of *Bacillus coagulans* and *Paenibacillus polymyxa* mixture in improving production and health of meat-type quails

**DOI:** 10.3389/fvets.2025.1639681

**Published:** 2025-09-24

**Authors:** Fayiz M. Reda, Mahmoud Alagawany, Ayman S. Salah, Layla A. Almutairi, Mohammed A. Alqahtani, Soha A. Alamoudi, Saleh Altuwaijri, Khaled A. El-Tarabily, Mohamed T. El-Saadony

**Affiliations:** ^1^Poultry Department, Faculty of Agriculture, Zagazig University, Zagazig, Egypt; ^2^Department of Animal Nutrition and Clinical Nutrition, Faculty of Veterinary Medicine, New Valley University, New Valley, Egypt; ^3^Department of Biology, College of Science, Princess Nourah bint Abdulrahman University, Riyadh, Saudi Arabia; ^4^Department of Biology, College of Science, King Khalid University, Abha, Saudi Arabia; ^5^Department of Biological Sciences, Faculty of Science, King Abdulaziz University, Jeddah, Saudi Arabia; ^6^Department of Pathology and Laboratory Diagnosis, College of Veterinary Medicine, Qassim University, Buraydah, Saudi Arabia; ^7^Department of Biology, College of Science, United Arab Emirates University, Al Ain, United Arab Emirates; ^8^Department of Agricultural Microbiology, Faculty of Agriculture, Zagazig University, Zagazig, Egypt

**Keywords:** antimicrobial activity, biochemical parameters, carcass characteristics, caecal microbiota, growth performance, immune response, organic poultry, probiotics

## Abstract

**Introduction:**

Despite the widespread interest in using *Bacillus* spp. as a probiotic in poultry diets, no evidence has been found to support the use of *Paenibacillus polymyxa* in the diet of Japanese quails. This study examined the effects of supplementing growing Japanese quail with a mixture of *Bacillus coagulans* and *P. polymyxa* (Bc+Pp) on their growth performance, antioxidative activity, immunological status, digestive enzymes, caecal microbiota, and blood chemistry.

**Methods:**

Two hundred 1-week-old meat-type quail chicks were divided into four groups at random; five pens, each containing ten birds. These birds were provided with a basic feed as a control group, or a feed diet treated with 0.5, 1.0, and 1.5 mg kg^−1^ of Bc+Pp mixture (1:1).

**Results:**

According to the findings, the growing quail's growth performance was significantly (*P* < 0.05) enhanced by supplementing the Bc+Pp mixture. Body weight and body weight gain were boosted significantly (*P* = 0.0002, *P* = 0.0003) by Bc+Pp mixture supplementation at 5 weeks and 1–5 weeks. In contrast, feed consumption showed a non-significant difference (*P* = 0.8082) with the treatments within 1–5 weeks. Moreover, the feed conversion ratio was significantly (*P* < 0.05) boosted (*P* = 0.0137) with the supplementation of the Bc+Pp mixture. Furthermore, Bc+Pp mixture supplementation provided a significant boost in carcass traits, especially liver, gizzard, and giblet percentage (*P* = 0.0112, *P* = 0.0976, and *P* = 0.0028). The current result showed a significant (*P* < 0.05) increase in total protein, albumin, and globulin with supplementation of the Bc+Pp mixture. Moreover, the treatment significantly (*P* < 0.05) reduced total cholesterol, triglycerides, and low-density lipoprotein. Superoxide dismutase, total antioxidant capacity, reduced glutathione, and glutathione peroxidase were significantly (*P* < 0.05) improved by supplementation of the Bc+Pp mixture. Furthermore, the digestive enzymes were significantly (*P* < 0.05) improved, and the total bacterial and lactic acid bacteria counts were significantly (*P* < 0.05) augmented, whereas the counts of *Salmonella* spp., *Escherichia coli*, total coliform, and *Enterococcus* spp. were significantly (*P* < 0.05) decreased with dietary bacterial mixture treatments.

**Discussion:**

In conclusion, supplementing growing Japanese quail with a mixture of Bc+Pp has a positive impact on their growth performance, antioxidative status, immunological response, digestive enzymes, and caecal microbiota.

## Introduction

Probiotics have emerged as promising alternatives to antibiotic growth promoters in poultry, addressing concerns about antibiotic residues and antimicrobial resistance (1–5). This shift supports safer and sustainable poultry production (6, 7). One hopeful substitute for improving growth performance and producing safe products simultaneously is the use of probiotic bacteria (7–9). Probiotics are recognized as living microorganisms that boost healthy gut microbiota, improve gut barrier function, stimulate the secretion of digestive enzymes, increase nutritional absorption, and enhance the performance of broilers and quails (10, 11).

Moreover, probiotic administration improved feed consumption and growth rate in Japanese quail (12, 13). A further study indicated that probiotic administration lowered triglyceride and cholesterol levels, hence improving the serum lipid profile of broilers (14). By generating antibacterial compounds, probiotics also lessen harmful bacteria by competitive exclusion (15, 16). As a probiotic, *Bacillus* spp. has advantages over other types, such as the capacity to withstand low pH in the intestine and high temperatures during the pelleting process (17).

Additionally, *Bacillus* spp. has demonstrated a positive impact on improving gut function via multiple processes, including the synthesis of antimicrobial substances, activating the intestinal immune system, and diminishing harmful microorganisms by competitive exclusion (18, 19). Furthermore, poultry's immune response and antioxidative state were enhanced by dietary *Bacillus* species (20).

Commercial probiotics in poultry feed are frequently derived from Gram-positive, spore-forming bacteria, *Bacillus coagulans*, and *Bacillus licheniformis* (21). Due to their protective protein coating and additional characteristics, they can endure environmental threats during the pelletization process, packaging, and treatment (21), as well as withstand gastric acidity, subsequently reaching the intestine, where they germinate and proliferate without generating enterotoxins (22).

*B. coagulans* is a homofermentative type that efficiently utilizes sugars and has lately been regarded as an innovative and safer probiotic. *B. coagulans* generates L-lactic acid as the predominant derivative of sugar fermentation, constituting around 97% of the fermented ingredients, whereas acetic acid and succinic acid are produced as small byproducts (22). *B. coagulans* preserves the intestinal mucus membrane barrier by boosting gut microbiota, facilitating the renewal of broiler intestinal mucosa, and bolstering congenital immunity (23).

Because of its huge biotechnology potential in sustainable agriculture and several industrial processes, *Paenibacillus polymyxa* (formerly *Bacillus polymyxa*) has garnered significant attention (24, 25) due to its production of two types of antimicrobial peptides [ribosomally-synthesized bacteriocins (Lantibiotics and pediocins), and non-ribosomally synthesized peptides (Cyclic cationic lipopeptides or cyclic non-cationic lipopeptides)] (25). Due to the beneficial effects of *P. polymyxa*, it could improve growth performance and body health status of quail chicks through improving immune response, antioxidative status, increasing beneficial bacterial count, and reducing pathogenic bacterial count (26).

No evidence exists concerning the benefits of *P. polymyxa* in the diet of Japanese quails, despite the considerable interest in *Bacillus* spp. as a probiotic in poultry nutrition. Previous studies examined the effects of *B. coagulans* or *P. polymyxa* individually as dietary supplements in poultry diets; however, no research has been conducted on the combined usage of these bacteria. It is hypothesized that the dietary addition of *B. coagulans* and *P. polymyxa* (Bc+Pp) mixture is expected to exert beneficial effects on the growing quails. Therefore, this study aimed to assess the impact of dietary supplementation with a combination of Bc+Pp on growth performance, carcass characteristics, liver and kidney function, antioxidative capacity, caecal microbiota, digestive enzyme activities, and immune responses in growing quails.

## Materials and methods

The present study adhered to the guidelines provided by the Local Experimental Animal Care Committee.

### Isolation, screening, and identification of bacterial isolates

The current research aimed to use and identify safe *Bacillus* isolates exhibiting antioxidant and antibacterial characteristics. This investigation involved the collection of isolates from fresh chicken feces obtained from poultry farm cages.

The fecal samples were transferred to sterile containers and conveyed to the microbiology laboratory within 24 h of collection. A 10-g fecal sample was homogenized in 90 ml of peptone buffer to achieve a 10^−1^ dilution, followed by the preparation of successive dilutions up to 10^−7^. After each dilution, the samples were inoculated into Luria-Bertani medium (LB, Lab M Limited, Lancashire, UK) and incubated at 37°C for 24 h. The most promising isolates were chosen due to their robust antibacterial efficacy against *Staphylococcus aureus, Salmonella typhi*, and *Pseudomonas aeruginosa* (27).

Among the tested isolates, 20 exhibited inhibition zones ranging from 20 to 30 mm. *B. coagulans* BcMT15 had the most substantial inhibitory zones, measuring 30 mm against *S. aureus*, 28 mm against *S. typhi*, and 26 mm against *P. aeruginosa*. Consequent to these findings, *B. coagulans* BcMT15 was chosen for subsequent use in this study.

The preliminary identification of *B. coagulans* BcMT15 was conducted utilizing established methodologies, including Gram staining, spore production, culture characteristics, pigmentation, and an array of biochemical and physiological assays, as detailed by Sneath (28). Matrix-assisted laser desorption ionization-time of flight mass spectrometry (MALDI-TOF MS) was employed to validate the identification (29). *B. coagulans* BcMT15 isolate exhibited 99% similarity to *B. coagulans* DSM 32016.

The acid resistance test was conducted to demonstrate the probiotic capabilities of *B. coagulans* BcMT15, utilizing a spectrophotometer to measure the optical density of each sample at 650 nm hourly in triplicate (30). The bile salt tolerance test was conducted (30). One ml bacterial culture was inoculated into 9 ml of LB broth at pH 2.5 and incubated at 37°C for 3 h. Following the incubation, a spectrophotometer was employed to assess the optical density of each sample at 650 nm. The absorbance level (A650) has been calibrated to 0.08 ± 0.05 to standardize bacterial counts.

Every acid-tolerant isolate underwent screening for bile tolerance. One hundred μl of overnight-cultivated bacterial culture was inoculated into newly produced LB broth containing 0.3% bile salts (Sigma-Aldrich Chemie GmbH, Taufkirchen, Germany). The vitality of bacteria in 0.3% bile was assessed by inoculating 100 μl of the bacterial sample onto the LB agar plate at time intervals of 0, 1, 2, 3, and 4 h. Plates devoid of bacterial colonies were classified as negative, while those containing colonies were classified as positive.

The acid tolerance and the survival rate were calculated using the following equation:


$$\begin{array}{l} Survival\;rate\;\left(\%\right)=\frac{OD\;after\;treatment}{OD\;before\;treatment}\times 100 \end{array}$$

*B. coagulans* BcMT15 showed a higher survival rate of 85.2 ± 3.1 at low pH and 78.6 ± 2.8 at 0.3% bile salt. The safety of the chosen bacterial isolates was assessed by evaluating their potential for antibiotic resistance and hemolytic activity. To assess hemolytic activity, each isolate was cultivated on blood agar plates and incubated for 48 h at 42°C. Isolates were cultured on nutrient agar medium (Lab M Limited) at a final concentration of 10^6^ colony-forming units (CFU) g^−1^ for antibiotic sensitivity testing. Standard antibiotic disks, including tetracycline, azithromycin, erythromycin, ceftriaxone, and gentamicin, were subsequently positioned on the medium. Results were documented during a 48 h incubation of the plates at 42°C.

The antioxidant and DNA-protective properties of the bacterial broth were assessed utilizing bacterial biosensors. The biosensor system employed *Escherichia coli* MG 1655 pRecA-lux (31), which incorporates luminescence genes regulated by a stress-inducible promoter and exhibits sensitivity to DNA damage. Following these evaluations, *B. coagulans* BcMT15 was identified as a promising probiotic strain for feed supplementation (32).

The safety assessment concentrated on hemolytic activity, mutagenicity (utilizing lux biosensors), and antibiotic resistance. The results indicated that none of the isolates had pro-mutagenic, hemolytic, or antibiotic-resistant characteristics. Of the 20 isolates evaluated, *B. coagulans* BcMT15 was selected for further investigation due to its superior combination of antimutagenic and antioxidant properties.

*Paenibacillus* isolates were obtained from fresh chicken fecal samples taken from a poultry farm, stored in sterile containers, and processed in the laboratory within 24 h. A 10-g fecal sample was homogenized in 90 ml of peptone buffer, then undergoing repeated dilutions from 10^−1^ to 10^−7^. Each dilution was inoculated into LB medium and incubated at 37°C for 24 h. Fifteen isolates were chosen for their significant antibacterial efficacy against *S. aureus, S. typhi*, and *P. aeruginosa*. Screening indicated that all 15 isolates produced inhibition zones of between 25 and 36 mm. *P. polymyxa* PpMT37 demonstrated the most extensive inhibitory zones, measuring 36 mm against *S. aureus*, 29 mm against *S. typhi*, and 32 mm against *P. aeruginosa*, and was chosen for further investigation as the most promising isolate.

The identification of *P. polymyxa* PpMT37 was conducted through standard methodologies, encompassing Gram staining, spore morphology, evaluation of cultural characteristics, pigment production, and an extensive array of biochemical and physiological tests, as outlined by Sneath (28). This identification was additionally corroborated using MALDI-TOF MS, in accordance with the protocols established by Bille et al. (29). The isolate *P. polymyxa* PpMT37 exhibited 99% similarity to *P. polymyxa* DSM 365.

The acid resistance of *P. polymyxa* PpMT37 isolate was assessed by inoculating cultures into LB broth (pH 2.5), incubating at 37°C for 3 h, and periodically measuring optical density at 650 nm. Bile salt tolerance was evaluated by inoculating overnight cultures into newly produced LB broth with 0.3% bile salts, followed by plating samples onto LB agar at different time intervals to ascertain viability (30). *P. polymyxa* PpMT37 exhibited a survival rate of 82.4 ± 2.9 at low pH and 76.3 ± 2.7 in the presence of 0.3% bile salt.

For safety evaluation, each isolate underwent hemolytic activity testing by culturing on blood agar at 42°C for 48 h, and antibiotic resistance assessment utilizing standard antibiotic disks (tetracycline, azithromycin, erythromycin, ceftriaxone, and gentamicin) on nutrient agar medium (Lab M Limited) at 10^8^ CFU g^−1^, with results documented after 48 h. The antioxidant and DNA-protective properties were assessed utilizing *E. coli* MG 1655 pRecA-lux biosensors, which react to DNA-damaging chemicals.

Of the 15 isolates assessed, *P. polymyxa* PpMT37 exhibited the most significant antimutagenic and antioxidant properties. All 15 *Paenibacillus* isolates showed an absence of pro-mutagenic, hemolytic, or antibiotic-resistant characteristics, while a few exhibited mild prooxidant activity. Biosensor tests were essential in finding isolates with enhanced antioxidant and antimutagenic properties, resulting in the selection of *P. polymyxa* PpMT37 for future investigation because of its exceptional bioactive profile.

### Antimicrobial activity of *B. coagulans* BcMT15 and *P. polymyxa* PpMT37

To evaluate the antibacterial activity of *B. coagulans* BcMT15 and *P. polymyxa* PpMT37 **(**1.5 × 10^8^ CFU ml^−1^) against different pathogenic Gram-positive and negative bacteria, four concentrations of 10%, 20%, 40%, and 80% were prepared. For each concentration, 8 mm disks were soaked for 30 min. The effectiveness of these disks was tested against several pathogenic bacteria that commonly infect poultry: *S. aureus, Streptococcus pyogenes, Listeria monocytogenes, S. typhi, E. coli*, and *Klebsiella pneumoniae*.

After the plates were inoculated with the bacteria, the saturated disks were placed on top. The plates were then incubated, and the resulting inhibition zones were measured in millimeters (33, 34).

### Experimental design

Two hundred growing quails, averaging 27.95 ± 0.37 g at 1 week of age, were randomly divided into four equal groups, each including five replications of ten quails. This study continued for 5 weeks and dietary treatment groups were as follows: first group: control (basal diet without bacterial supplementation); second group (0.5 mg Bc+Pp; basal diet + 0.25 mg *B. coagulans* + 0.25 mg *P. polymyxa* kg diet^−1^); third group (1.0 mg Bc+Pp; basal diet + 0.5 mg *B. coagulans* + 0.5 mg *P. polymyxa* kg diet^−1^); and the fourth group (1.5 g Bc+Pp; basal diet + 0.75 mg *B. coagulans* + 0.75 mg *P. polymyxa* kg diet^−1^) and the concentrations of *B. coagulans* and *P. polymyxa* were 1.0 × 10^6^ CFU g^−1^ and 1.5 × 10^8^ CFU ml^−1^, respectively.

The standards outlined in (35) were utilized to design the basal diet to meet the requirements of quail throughout the quail phase (Table 1). A typical cage of 50 × 30 × 50 cm3 was employed for the rearing of quails. Water and nourishment were provided *ad libitum*.

**Table 1:** Ingredients and nutrient contents of the basal diet of growing Japanese quail.

**Parameters**	**(g kg^−1^)**
**Ingredient**	
Maize (8.5%)	518.0
Soybean meal (44%)	367.0
Maize gluten meal (62%)	52.1
Soybean oil	29.0
Limestone	7.0
Di-calcium phosphate	16.5
Salt	3.0
Vitamin premix^*^	3.0
L-Lysine	1.3
DL-Methionine	1.1
Choline chloride	2.0
Total	1,000
**Calculated composition**	
Metabolizable energy (MJ kg^−1^)	12.5
Crude protein (g kg^−1^)	240
Calcium (g kg^−1^)	8.0
Non-phytate phosphorus (g kg^−1^)	4.5
Lysine (g kg^−1^)	13.0
Total sulfur amino acids (g kg^−1^)	9.2

^*^Vitamin premix per kg of diet: vitamin A, 12,000 IU; vitamin D3, 5,000 IU; vitamin E, 130.0 mg; vitamin K3, 3.605 mg; vitamin B1 (thiamin), 3.0 mg; vitamin B2 (riboflavin), 8.0 mg; vitamin B6, 4.950 mg; vitamin B12, 17.0 mg; niacin, 60.0 mg; D-biotin, 200.0 mg; calcium D-pantothenate, 18.333 mg; folic acid, 2.083 mg; manganese, 100.0 mg; iron, 80.0 mg; zinc, 80.0 mg; copper, 8.0 mg; iodine, 2.0 mg; cobalt, 500.0 mg; and selenium, 150.0 mg.

### Determination of growth performance and carcass characteristics

Quail weights were recorded at 1, 3, and 5 weeks of age to calculate body weight and body weight gain. During the trial, feed intake and feed conversion ratio were calculated. At week 5, six birds from each treatment group were randomly selected and euthanized for carcass analysis.

### Assessment of blood biochemistry, digestive enzyme activity, immunological markers, and antioxidant activities in quails

Blood samples were obtained from the slaughtered birds directly into a tube containing the anticoagulant ethylene diamine tetraacetic acid (EDTA, Sigma-Aldrich). The serum was obtained using centrifugation of the blood sample at 4,000 rpm for 10 min, as recommended by Zhou et al. (36). The serum was transferred into a sterile tube, which was thereafter sealed and kept at −20°C until utilized.

The biochemical parameters of alanine aminotransferase, aspartate transaminase, lactate dehydrogenase, urea, creatinine, total cholesterol, high-density lipoprotein, low-density lipoprotein, and very low-density lipoprotein were quantified utilizing an automated analyzer and a Biodiagnostic commercial kit (Biodiagnostic, Giza, Egypt) in accordance with the manufacturer's guidelines (36). Creatinine, uric acid, and urea levels were measured using commercially available Quimica Clinica Aplicada kits (Quimica Clinica Aplicada, Tarragona, Spain).

Total protein was quantified with the Biuret method as described by Armstrong and Carr (37). Albumin concentrations were determined using a calorimetric technique (38). Globulin concentrations were determined by deducting albumin concentrations from total protein concentrations. Amylase activity was determined by the Somogyi method (39), and the technique recommended by Tietz and Fiereck (40) was used for the lipase enzyme. The methods described by Lynn and Clevette-Radford (41) were used to measure protease activity. Immunoglobulins were evaluated using enzyme-linked immunosorbent assay (ELISA) as described by Gao et al. (42).

### Assessment of caecal microbial counts

To obtain the caecal content, three birds from each replication were slaughtered. Upon separating the caeca, the contents were meticulously collected in sterile cups to guarantee aseptic handling. The samples were preserved at 4°C until the quantification of the microbial population. A dilution factor of 10^−6^ was attained by a tenfold successive dilution of caecal material. Aliquots of 0.2 ml were dispensed using a sterile glass rod in sterile plastic Petri plates with a 90 mm diameter over different general-purpose and selective agar medium.

The selected organisms for enumeration and the corresponding media utilized were as follows: (i) total aerobic bacteria on nutrient agar medium (Lab M Limited, Product Code: LAB008); (ii) total yeasts and molds on Sabouraud dextrose agar (HiMedia Laboratories Pvt. Ltd., Mumbai, India, Product Code: MH063); (iii) *E. coli* on eosin methylene blue agar (Lab M Limited, Product Code: LAB061); (iv) total coliforms on MacConkey agar medium (Lab M Limited, Product Code: LAB045); (v) *Salmonella* spp. on xylose lysine decarboxylase agar (XLD agar; Lab M Limited, Product Code: LAB032); *Enterococcus* spp. on *Enterococcus* agar (HiMedia Laboratories, Product Code: MH2077); and (vi) lactic acid bacteria on MRS agar (Lab M Limited, Product Code: LAB223).

Following 20 min of drying in a laminar flow cabinet, the plates were incubated at 30°C in darkness for 3 days to enumerate total aerobic bacteria, total coliforms, *E. coli, Salmonella* spp., *Enterococcus*, and lactic acid bacteria. The plates were incubated in the dark at 28°C for 6 days to enumerate total yeasts and molds.

Six plates were made for each dilution for every sample and replication. Population densities were assessed by quantifying CFU per gram of dry caecal weight and thereafter expressing the results as log_10_ CFU (43, 44).

### Statistical analysis

In accordance with Steel and Torrie (45), all microbiological tests were conducted in triplicate. Statistical analysis was performed using one-way analysis of variance (ANOVA). All statistical analyses were carried out using IBM SPSS 23 Statistics for Mac OS (Armonk, NY, USA). The least significant difference (LSD) test was used to compare all tested means (treatments) with a probability of *P* < 0.05.

The statistical model applied was: Y_ij_ = μ + T_i_ + e_ij_ where: Y_ij_ = an observation; μ = overall mean; T_i_ = the fixed effect of probiotic treatments; and e_ij_ = Random error. The pen served as the experimental unit for growth performance and carcass features, whereas the individual bird was designated as the experimental unit for biochemical, microbiological, enzymatic, and immunological parameters.

## Results

### Isolation, screening, and identification of bacterial isolates

Of the 20 isolates evaluated, *B. coagulans* BcMT15 was selected for further investigation due to its superior combination of antibacterial, antimutagenic, and antioxidant properties (Supplementary Tables S1–S4). Furthermore, out of the 15 isolates assessed, *P. polymyxa* PpMT37 exhibited the most significant antibacterial, antimutagenic, and antioxidant properties and was selected for further investigation (Supplementary Tables S5–S8).

### Antibacterial activity of *B. coagulans* BcMT15 and *P. polymyxa* PpMT37

Table 2 provides a comparative assessment of the antibacterial efficacy of *B. coagulans* BcMT15 and *P. polymyxa* PpMT37 against six pathogenic bacterial strains. *P. polymyxa* PpMT37 consistently showed superior antibacterial efficacy compared to *B. coagulans* BcMT15 against all evaluated pathogens (Supplementary Figure S1).

**Table 2:** Antibacterial activity of *Bacillus coagulans* BcMT15 and *Paenibacillus polymyxa* PpMT37 against pathogenic Gram-positive and Gram-negative bacteria.

**Pathogenic bacteria**	*Bacillus coagulans* BcMT15^*^	*Paenibacillus polymyxa* PpMT37^*^
*Staphylococcus aureus*	30.0 ± 1.5	34.2 ± 1.7
*Streptococcus pyogenes*	28.5 ± 1.4	32.8 ± 1.6
*Listeria monocytogenes*	27.2 ± 1.3	31.5 ± 1.5
*Salmonella typhi*	25.9 ± 1.2	30.1 ± 1.4
*Escherichia coli*	24.8 ± 1.1	29.7 ± 1.4
*Klebsiella pneumoniae*	23.6 ± 1.0	28.3 ± 1.3

^*^Inhibition zone diameter in mm. Mean values expressed as mean ± standard error. The inhibition zones for the concentration of (80%).

*B. coagulans* BcMT15 (80%) had reduced, but nevertheless, notable, action, demonstrating the most substantial inhibition zone against *S. aureus* (30.0 ± 1.5 mm) and the least against *K. pneumoniae* (23.6 ± 1.0 mm; Table 2 and Supplementary Figure S1). In contrast, the most significant inhibition zone for *P. polymyxa* PpMT37 (80%) was recorded against *S. aureus* (34.2 ± 1.7 mm), followed by *S. pyogenes* (32.8 ± 1.6 mm), *L. monocytogenes* (31.5 ± 1.5 mm), *S. typhi* (30.1 ± 1.4 mm), *E. coli* (29.7 ± 1.4 mm), and *K. pneumoniae* (28.3 ± 1.3 mm; Table 2 and Supplementary Figure S1).

These findings indicated that *P. polymyxa* PpMT37 was more efficacious in suppressing the development of these pathogenic bacteria compared to *B. coagulans* BcMT15, as seen by the larger inhibition zones (Supplementary Figure S1). The uniformity of the results, indicated by the minimal standard deviations, emphasizes the dependability of the findings. This comparative study underscores the potential of *P. polymyxa* PpMT37 as a viable candidate for further investigation.

### Effect of *B. coagulans* and *P. polymyxa* on the productive performance of Japanese quails

Table 3 illustrates the effects of a dietary Bc+Pp combination on the productive performance of meat-type quail. The results indicated a significant (*P* < 0.05) improvement in body weight throughout the trial duration, particularly at 3 and 5 weeks. The fourth group, administered 1.5 mg kg^−1^ of the bacterial combination, exhibited a significant (*P* < 0.05) rise in body weight (103.83 g) at the 3-week mark (*P* = 0.0077). The third group administered 1.0 mg kg^−1^ of bacterial combination had a significant (*P* < 0.05) increase (*P* = 0.0002) in body weight (230.38 g) at 5 weeks of age (Table 3).

**Table 3:** Effect of dietary *Bacillus coagulans* and *Paenibacillus polymyxa* mixture on productive performance of growing quail.

**Parameters**	***Bacillus coagulans*** **and** ***Paenibacillus polymyxa*** **levels (mg kg**^**−1**^ **diet)**				**SEM**	***P* value**
	**0**	**0.5**	**1.0**	**1.5**		
**Body weight (g)**						
1 week	27.98a	27.90a	27.99a	27.91a	0.370	0.9974
3 weeks	94.90a	105.34b	103.67b	103.83b	1.503	0.0077
5 weeks	197.95c	223.00a	230.38a	213.16b	2.672	0.0002
**Body weight gain (g day** ^−1^ **)**						
1–3 weeks	4.78a	5.53b	5.41b	5.42b	0.118	0.0116
3–5 weeks	7.36c	8.40ab	9.05a	7.81bc	0.190	0.0027
1–5 weeks	6.07c	6.97a	7.23a	6.62b	0.101	0.0003
**Feed intake (g day** ^−1^ **)**						
1–3 weeks	15.19a	15.01a	14.82a	15.04a	0.490	0.9634
3–5 weeks	20.93a	19.60a	20.95a	20.43a	0.593	0.4560
1–5 weeks	18.06a	17.30a	17.88a	17.73a	0.541	0.8082
**Feed conversion ratio (g feed g gain** ^−1^ **)**						
1–3 weeks	3.18a	2.71a	2.74a	2.78a	0.084	0.0517
3–5 weeks	2.85a	2.34b	2.31b	2.62ab	0.099	0.0230
1–5 weeks	2.98a	2.48b	2.47b	2.68b	0.076	0.0137

Mean values expressed as mean ± standard error. Values with the same letter within a row are not significantly (*P*>0.05) different according to the least significant difference (LSD) test.

Moreover, the bacterial mixture supplementation significantly (*P* < 0.05) enhanced (*P* = 0.0003) body weight gain throughout the trial period (weeks 1–5), with the third group (1.0 mg kg^−1^ Bc+Pp) demonstrating a superior body weight gain of 7.23 g from weeks 1 to 5 compared to the other groups and the control (Table 3). Furthermore, the bacterial combination treatment did not significantly (*P*>0.05) influence feed consumption during the study periods (weeks 1–5). The feed conversion ratio was significantly (*P* < 0.05) influenced by treatments throughout the trial duration, with the third group (1.0 mg kg^−1^ Bc+Pp) demonstrating superior values (*P* = 0.0230, *P* = 0.0137) during weeks 3–5 and 1–5, recording values of 2.31 and 2.47, respectively (Table 3).

### Effect of *B. coagulans* and *P. polymyxa* on the carcass traits of Japanese quails

Table 4 displays the results of the interaction between the dietary Bc+Pp combination and the carcass characteristics of meat-type quails. The findings showed that there was no significant difference in the percentage of the carcass, gizzard, and heart (*P* = 0.2130, *P* = 0.0976, *P* = 0.3816) among all treatments (Table 4). Furthermore, the treatment with the bacterial combination for developing quail showed a significant (*P* < 0.05) rise in the percentage of the liver, giblets, and dressing (*P* = 0.0112, *P* = 0.0028, *P* = 0.0685). Additionally, the second group that was supplemented with a meal containing 0.5 mg kg^−1^ of the bacterial mixture showed an increased liver. As a result, the third group (1.0 mg kg^−1^ Bc+Pp) demonstrated an increase in the percentage of giblets and dressing (Table 4).

**Table 4:** Effect of dietary *Bacillus coagulans* and *Paenibacillus polymyxa* mixture on carcass traits and relative organ weights of growing quail.

**Parameters**	***Bacillus coagulans*** **and** ***Paenibacillus polymyxa*** **levels (mg kg**^**−1**^ **diet)**				**SEM**	***P* value**
	**0**	**0.5**	**1.0**	**1.5**		
Carcass (%)	77.18a	78.46a	79.96a	78.76a	0.831	0.2130
Liver (%)	2.17b	2.58a	2.46a	2.51a	0.066	0.0112
Gizzard (%)	2.33a	2.42a	2.71a	2.29a	0.110	0.0976
Heart (%)	1.11a	1.00a	1.13a	0.98a	0.069	0.3816
Giblets (%)	5.61c	6.00b	6.30a	5.77bc	0.082	0.0028
Dressing (%)	82.79a	84.46a	86.26a	84.53a	0.749	0.0685

Mean values expressed as mean ± standard error. Values with the same letter within a row are not significantly (*P*>0.05) different according to the least significant difference (LSD) test.

### Effect of *B. coagulans* and *P. polymyxa* on biochemical parameters of Japanese quails

Table 5 illustrates the impact of a dietary combination of *B. coagulans* and *P. polymyxa* on the hepatic and renal functions of developing quail. The results indicated a significant (*P* < 0.05) rise in total protein, albumin, and globulin (*P* = 0.0027, *P* = 0.0283, and *P* = 0.0002) following bacterial combination treatments, with the third group (1.0 mg kg^−1^ Bc+Pp) exhibiting elevated values (4.39, 2.18, 2.22 g dl^−1^; Table 5).

**Table 5:** Effect of dietary *Bacillus coagulans* and *Paenibacillus polymyxa* mixture on liver and kidney functions of growing quail.

**Parameters**	***Bacillus coagulans*** **and** ***Paenibacillus polymyxa*** **levels (mg kg**^**−1**^ **diet)**				**SEM**	***P* value**
	**0**	**0.5**	**1.0**	**1.5**		
TP (g dl^−1^)	2.56c	3.31b	4.39a	3.79ab	0.222	0.0027
ALB (g dl^−1^)	1.46b	1.72ab	2.18a	1.96a	0.130	0.0283
GLOB (g dl^−1^)	1.11c	1.59b	2.22a	1.83b	0.092	0.0002
A/G ratio	1.33a	1.08b	0.98b	1.07b	0.032	0.0009
ALT (IU L^−1^)	18.58a	13.41*b*	9.38*c*	14.32*b*	0.851	0.0007
AST (IU L^−1^)	196.73a	135.64b	203.20a	223.30a	9.366	0.0012
LDH (IU L^−1^)	216.53a	148.95c	179.35b	172.05b	6.939	0.0009
Creatinine (mg dl^−1^)	0.69a	0.48a	0.50a	0.44a	0.083	0.2189
Urea (mg dl^−1^)	1.53a	1.16b	1.14b	1.57a	0.069	0.0038
Uric acid (mg dl^−1^)	8.07a	6.44ab	4.99b	5.93b	0.504	0.0162

TP, total protein; ALB, albumin; GLOB, globulin; A/G, albumin/globulin ratio; ALT, alanine aminotransferase; AST, aspartate aminotransferase; LDH, lactate dehydrogenase.

Mean values expressed as mean ± standard error. Values with the same letter within a row are not significantly (*P*>0.05) different according to the least significant difference (LSD) test.

The findings indicated a significant (*P* < 0.05) reduction in liver alanine aminotransferase, aspartate transaminase, and lactate dehydrogenase following bacterial combination treatments (*P* = 0.0007, *P* = 0.0012, and *P* = 0.0009), with the third group (1.0 mg kg^−1^ Bc+Pp) exhibiting decreased alanine aminotransferase levels (9.38 IU L^−1^; Table 5). Conversely, the second group (0.5 mg kg^−1^ Bc+Pp) had reduced aspartate transaminase and lactate dehydrogenase levels (135.64, 148.95) IU L^−1^ (Table 5).

The results also indicated a non-significant reduction in creatinine levels (*P* = 0.2189) following bacterial combination therapy, with the fourth group exhibiting decreased levels of 0.44 mg dl^−1^. The findings indicated a significant (*P* < 0.05) reduction in urea and uric acid levels (*P* = 0.0038, *P* = 0.0162), with the third group (1.0 mg kg^−1^ Bc+Pp) exhibiting decreased concentrations of 1.14 and 4.99 mg dl^−1^, respectively (Table 5).

### Effect of *B. coagulans* and *P. polymyxa* on the lipid profile of Japanese quails

Table 6 presents the effects of the dietary Bc+Pp combination on the lipid profile of developing quail. The results indicated a significant (*P* < 0.05) decrease in total cholesterol, triglycerides, low-density lipoprotein, and very low-density lipoprotein (*P* < 0.0001, *P* = 0.0003, *P* < 0.0001, and *P* = 0.0003), with the second group treated with 0.5 mg kg^−1^ of the bacterial mixture exhibiting reduced levels of 184.35, 138.00, 92.53, and 27.60 mg dl^−1^, respectively (Table 6). The results also demonstrated that the third group (1.0 mg kg^−1^) significantly (*P* < 0.05) elevated high-density lipoprotein levels (68.10 mg dl^−1^) with bacterial mixture supplementation (Table 6).

**Table 6:** Effect of dietary *Bacillus coagulans* and *Paenibacillus polymyxa* mixture on lipid profile of growing quail.

**Parameters**	***Bacillus coagulans*** **and** ***Paenibacillus polymyxa*** **levels (mg kg**^**−1**^ **diet)**				**SEM**	***P* value**
	**0**	**0.5**	**1.0**	**1.5**		
TC (mg dl^−1^)	270.22a	184.35c	206.09b	207.30b	5.649	< 0.0001
TG (mg dl^−1^)	208.01a	138.00c	181.85b	200.10ab	6.567	0.0003
HDL (mg dl^−1^)	52.50c	64.23ab	68.10a	59.30bc	2.499	0.0126
LDL (mg dl^−1^)	176.12a	92.53b	101.63b	107.99b	6.030	< 0.0001
VLDL (mg dl^−1^)	41.60a	27.60c	36.37b	40.02ab	1.313	0.0003

TC, total cholesterol; TG, triglycerides; HDL, high-density lipoprotein; LDL, low-density lipoprotein; VLDL, very low-density lipoprotein.

Mean values expressed as mean ± standard error. Values with the same letter within a row are not significantly (*P*>0.05) different according to the least significant difference (LSD) test.

### Effect of *B. coagulans* and *P. polymyxa* on antioxidants and immunity status of Japanese quails

Table 7 illustrates the impact of a dietary combination of *B. coagulans* and *P. polymyxa* on the immunity and antioxidant levels of developing quail. The findings demonstrated a significant (*P* < 0.05) enhancement in antioxidant status following bacterial mixture supplementation, with the third group (1.0 mg kg^−1^ Bc+Pp) exhibiting significant (*P* < 0.05) increases in superoxide dismutase, reduced glutathione, and glutathione peroxidase levels (*P* = 0.0003, *P* = 0.0002, *P* = 0.0021; 0.41 U ml^−1^, 0.35 ng ml^−1^, 0.54 ^−1^, respectively; Table 7).

**Table 7:** Effect of dietary *Bacillus coagulans* and *Paenibacillus polymyxa* mixture on immunity and antioxidants of growing quail.

**Parameters**	***Bacillus coagulans*** **and** ***Paenibacillus polymyxa*** **levels (mg kg**^**−1**^ **diet)**				**SEM**	***P* value**
	**0**	**0.5**	**1.0**	**1.5**		
**Antioxidants**						
SOD (U ml^−1^)	0.25c	0.34b	0.41a	0.30b	0.013	0.0003
MDA (nmol ml^−1^)	0.49a	0.22bc	0.12c	0.28b	0.038	0.0013
TAC (ng ml^−1^)	0.17c	0.26c	0.34b	0.44a	0.026	0.0006
GSH (ng ml^−1^)	0.13c	0.23b	0.35a	0.30a	0.018	0.0002
GPX (mU ml^−1^)	0.22b	0.34b	0.54a	0.49a	0.038	0.0021
**Immunity**						
IgM (mg dl^−1^)	0.58b	1.08a	0.85ab	0.91a	0.085	0.0203
IgY (mg dl^−1^)	0.48a	0.52a	0.69a	0.48a	0.088	0.3562
IgA (mg dl^−1^)	0.69b	0.89ab	1.13a	1.07a	0.079	0.0175
Complement 3 (mg dl^−1^)	127.65c	174.13b	209.23a	187.00ab	9.512	0.0019
Lysozyme (U ml^−1^)	0.14c	0.24b	0.28ab	0.32a	0.020	0.0015

SOD, superoxide dismutase; MDA, malondialdehyde; TAC, total antioxidant capacity; GSH, reduced glutathione; GPX, glutathione peroxidase; IgM, immunoglobulin M, IgY, immunoglobulin Y, IgA, immunoglobulin A.

Mean values expressed as mean ± standard error. Values with the same letter within a row are not significantly (*P*>0.05) different according to the least significant difference (LSD) test.

Moreover, the results indicated that the third group (1.0 mg kg^−1^ Bc+Pp) exhibited a significant (*P* < 0.05) reduction in malondialdehyde levels (0.12 nmol ml^−1^, *P* = 0.0013), while the fourth group (1.5 mg kg^−1^ Bc+Pp) had a significant (*P* < 0.05) rise in total antioxidant capacity concentration (0.44 ng ml^−1^, *P* = 0.0006; Table 7).

The findings also demonstrated a significant (*P* < 0.05) rise in IgM and IgA (*P* = 0.0203, and *P* = 0.0175), with the second group (0.5 mg kg^−1^ Bc+Pp) exhibiting a heightened IgM concentration (1.08 mg dl^−1^) and the third group (1.0 mg kg^−1^ Bc+Pp) showing elevated IgA levels (1.13 mg dl^−1^; Table 7). Furthermore, the introduction of a bacterial combination to the food of developing quail resulted in a non-significant elevation in IgY levels (*P* = 0.3562), with the third group exhibiting elevated levels (1.13 mg dl^−1^; Table 7).

Moreover, the introduction of a bacterial combination to the growth diet markedly enhanced levels of complement 3 and lysozymes (*P* = 0.0019, *P* = 0.0015). The third group (1.0 mg kg^−1^ Bc+Pp) exhibited elevated complement 3 levels (209.23 μ ml^−1^), while the fourth group (1.5 mg kg^−1^ Bc+Pp) had an increased lysozyme level (0.32 μ ml^−1^; Table 7).

### Effect of *B. coagulans* and *P. polymyxa* on the status of digestive enzymes of Japanese quails

The impact of a dietary combination of *B. coagulans* and *P. polymyxa* on the digestive enzymes of developing quail is presented in Table 8. The results indicated a significant (*P* < 0.05) increase in amylase, lipase, and protease enzyme levels (*P* = 0.0005, *P* = 0.0013, and *P* = 0.0006) due to bacterial mixture supplementation, with the fourth group (1.5 mg kg^−1^ Bc+Pp) exhibiting elevated amylase levels (16.22 U L^−1^). The third group (1.0 mg kg^−1^ Bc+Pp) had elevated levels of lipase and protease enzymes (12.84, 1.56 U L^−1^) compared to the control and other groups (Table 8).

**Table 8:** Effect of dietary *Bacillus coagulans* and *Paenibacillus polymyxa* mixture on digestive enzymes of growing quail.

**Parameters**	***Bacillus coagulans*** **and** ***Paenibacillus polymyxa*** **levels (mg kg**^**−1**^ **diet)**				**SEM**	***P* value**
	**0**	**0.5**	**1.0**	**1.5**		
Amylase (U L^−1^)	4.51b	7.09b	13.99a	16.22a	1.130	0.0005
Lipase (U L^−1^)	3.49b	6.38b	12.84a	10.46a	1.047	0.0013
Protease (U L^−1^)	0.59c	0.97b	1.56a	1.10b	0.092	0.0006

Mean values expressed as mean ± standard error. Values with the same letter within a row are not significantly (*P*>0.05) different according to the least significant difference (LSD) test.

### Effect of *B. coagulans* and *P. polymyxa* on caecal bacterial count of Japanese quails

The effect of a dietary combination of *B. coagulans* and *P. polymyxa* on the caecal bacterial count (Log_10_ CFU g^−1^) in developing quail is presented in Table 9. The results demonstrated a significant (*P* < 0.05) rise (*P* = 0.0002) in total bacterial count due to bacterial mixture supplementation, with the third group (1.0 mg kg^−1^ Bc+Pp) exhibiting elevated levels (Table 9). Furthermore, the supplementation of bacterial mixture significantly (*P* < 0.05) reduced the overall counts of yeasts and molds, as well as *E. coli*, total coliforms, *Salmonella* spp., and *Enterococcus* spp. Conversely, there was a significant (*P* < 0.05) difference in the counts of lactic acid bacteria, with the third group (1.0 mg kg^−1^ Bc+Pp) exhibiting enhanced outcomes (Table 9).

**Table 9:** Effect of dietary *Bacillus coagulans* and *Paenibacillus polymyxa* mixture on caecal bacterial count (Log_10_ CFU g^−1^) of growing quail.

**Microbial counts**	***Bacillus coagulans*** **and** ***Paenibacillus polymyxa*** **levels (mg kg**^**−1**^ **diet)**				**SEM**	***P* value**
	**0**	**0.5**	**1.0**	**1.5**		
Total bacteria	4.78b	5.47a	5.72a	5.49a	0.079	0.0002
Total yeasts and molds	5.53a	4.93c	4.66d	5.17b	0.058	< 0.0001
*Escherichia coli*	5.46a	5.10b	5.00bc	4.84c	0.129	0.0011
Total coliform	5.65a	5.21b	5.03b	5.43a	0.065	0.0006
*Salmonella* spp.	3.56a	1.93b	1.7bc	1.48c	0.087	< 0.0001
Lactic acid bacteria	4.40c	5.11b	5.49a	5.45a	0.113	< 0.0001
*Enterococcus* spp.	5.53a	4.55b	4.40b	4.02c	0.025	< 0.0001

Mean values expressed as mean ± standard error. Values with the same letter within a row are not significantly (*P*>0.05) different according to the least significant difference (LSD) test.

## Discussion

### Growth performance of meat-type quail

*Bacillus* strains enhance the productive performance of hens (46). Nevertheless, information regarding the advantageous effects of *P. polymyxa* on poultry is limited (9). Furthermore, no research exists concerning the influence of the Bc+Pp on Japanese quail. Consequently, our research sought to examine the advantageous impact of the Bc+Pp on the diets of meat-type quails. Wu et al. (9) previously investigated the advantageous effects of *P. polymyxa* on broiler chickens, revealing that those fed diets supplemented with *P. polymyxa* showed significant (*P* < 0.05) improvements in overall health by enhancing gut barrier function, decreasing cell apoptosis, and increasing antioxidative capacity (9). In the same context, economic efficiency of broiler chickens was improved by using *P. polymyxa* in their diet, which also improved their growth rate, carcass dressing and the composition of the gut microbiota (47). Similarly, *P. polymyxa* 10 slightly improved the growth performance of broiler chickens during the starter phase (48).

Probiotics made from the bacteria *Paenibacillus xylanexedens ysm1* have recently been shown to increase broiler chickens productivity (8, 49). The addition of *Bacillus subtilis* spores to the diet of Japanese quail reduced their feed conversion ratio and increased their body weight and body weight gain (50). Additionally, quail health and development performance were also improved by these probiotics (11).

Moreover, chickens receiving *B. coagulans* diets showed a significant (*P* < 0.05) increase in body weight, body weight gain to feed intake ratio from day 15 to 21 (51). The incorporation of a dietary blend of *B. coagulans* and *P. polymyxa* improved body weight and body weight gain in meat-type quail. The highest body weight and body weight gain were seen in the 1.0 mg kg^−1^ dietary group. Moreover, the administration of a *B. coagulans* and *P. polymyxa* combination enhanced the feed conversion ratio, with the most effective dosage being 1 mg kg^−1^ of feed. These results correspond with the findings of Seifi et al. (52). Their results showed that administering single-dose probiotics improves quails' body weight increase and feed intake. Gupta et al. (53), found that dietary interventions with probiotics improved the performance of Japanese quail. The use of probiotics improves growth performance by increasing beneficial gut flora. Enhancing gut barrier function enhances the release of digestive enzymes, hence augmenting nutrient absorption and facilitating body weight and body weight gain (12, 54).

Additionally, Flores et al. (55) demonstrated that broilers administered *Bacillus* strains exhibited enhanced feed intake, feed efficiency, and body weight gain. The enhancement of feed efficiency in groups treated with a mixture of *B. coagulans* and *P. polymyxa* may result from the promotion of gut health through the augmentation of beneficial microflora, the suppression of harmful bacteria via competitive elimination, and the synthesis of antibacterial agents (16), or from increased nutrient digestibility resulting in improved feed conversion ratio (56). Conversely, several studies have revealed that the incorporation of probiotics in broiler diets does not affect productive efficiency (20, 57). The discrepancies in the results may arise from variations in experimental methodologies, including probiotic strains, doses, avian age, and breed (58).

In the current study, the inclusion of a *B. coagulans* and *P. polymyxa* mixture at 1 mg kg^−1^ in the diet enhanced productive performance qualities and increased giblet percentage in growing quail, with no significant variations observed in carcass features. The observed increase in giblet percentage, particularly liver weight, in quails supplemented with *B. coagulans* and *P. polymyxa* may reflect a physiological adaptation associated with enhanced growth performance and improved nutrient utilization (Table 3), as well as improved liver function (Table 5).

Probiotics have been reported to stimulate hepatic function through the upregulation of protein synthesis, lipid metabolism, and detoxification pathways, which could result in slight organ enlargement within a healthy range (51). The inclusion of probiotics in broiler diets did not result in substantial variations in dressing and carcass percentages (59) or carcass yield (51). Conversely, Kaushal et al. (60) reported that the inclusion of probiotics in the broiler's diet positively influenced dressing yield.

### Blood biochemical parameters

The current study demonstrated that a mixture of *B. coagulans* and *P. polymyxa* significantly (*P* < 0.05) elevated total protein and globulin concentration while exhibiting no variation in albumin, thereby resulting in a decreased albumin/globulin ratio. These findings are consistent with prior research on meat-type quails (61, 62) and poultry (63). The observed impacts may result from enhanced dietary protein utilization in groups supplemented with a mixture of *B. coagulans* and *P. polymyxa* achieved by inhibiting pathogen proliferation, which diminishes protein degradation into nitrogen, enhances dietary protein metabolism and expands the surface area for utilization of digested feed (63), or by augmenting the absorption power of the intestinal villi (64).

In our investigation, the dietary addition of a mixture of *B. coagulans* and *P. polymyxa* decreased blood aspartate aminotransferase and lactate dehydrogenase levels, particularly in the 0.5 mg kg^−1^ diet group. The reduction in aspartate aminotransferase corresponds with the results of Kasmani et al. (61) and Abramowicz et al. (65), suggesting that the combination of Bc+Pp provides a protective role in the liver by reducing pathogen translocation, thereby decreasing serum transaminase levels (66).

Lactate dehydrogenase is acknowledged as an indicator of cellular toxicity (67), and a decrease in lactate dehydrogenase levels in groups treated with a mixture of *B. coagulans* and *P. polymyxa* suggests a reduction in cellular damage (65). Concerning renal function, there was no notable reduction in creatinine levels, whereas there was a significant (*P* < 0.05) decrease in urea and uric acid attributable to dietary supplementation with a mixture of *B. coagulans* and *P. polymyxa*. Our findings support a previous study (20) suggesting that dietary probiotics reduce urea and creatinine levels by improving protein digestion in Japanese quail.

Our current results demonstrated that the administration of *B. coagulans* and *P. polymyxa* mixture in the diet of meat-type quails resulted in a hypolipidemic effect, as indicated by significantly (*P* < 0.05) reduced levels of total cholesterol, triglycerides, low-density lipoprotein, and very low-density lipoprotein. The 1.0 mg kg^−1^ feed had the highest efficacy among the supplemented groups. The reduction in cholesterol and triglycerides due to probiotic inclusion corresponds with prior studies on developing quail and chicken. In contrast, the administration of *P. polymyxa* had no significant effect on cholesterol and triglyceride levels in fowl (9).

The hypocholesterolemic effect of the *B. coagulans* and *P. polymyxa* mixture may arise from the modulation of lipid metabolism through the deconjugation of bile salts and the quantity of cholesterol absorbed from the gastrointestinal tract, as the co-precipitation of intestinal cholesterol interacts with deconjugated bile salts, thereby inhibiting the synthesis of the intestinal cholesterol transporter (68). The increase of high-density lipoprotein and the decrease of low-density lipoprotein and very low-density lipoprotein in the *B. coagulans* and *P. polymyxa* mixture-treated groups correspond with earlier studies (50, 65). Ognik et al. (69) reported that the dietary inclusion of probiotics reduces oxidative processes inside cells, resulting in lower levels of total cholesterol, triglycerides, and low-density lipoprotein.

### Antioxidative capacity and immune responses

The current findings indicated that a dietary Bc+Pp enhanced the antioxidant capacity of meat-type quail by considerably reducing malondialdehyde levels and increasing superoxide dismutase and total antioxidant capacity concentrations. The optimal dosage was 1.0 mg kg^−1^ diet. The introduction of *P. polymyxa* in broiler diets enhanced antioxidant capability by reducing malondialdehyde levels and elevating reduced glutathione and glutathione peroxidase activity (9). The inclusion of dietary *Bacillus* enhanced the actions of superoxide dismutase and glutathione peroxidase in poultry. The findings align with those of Abdel-Moneim et al. (50), who demonstrated that the incorporation of *Bacillus*-based probiotics in the diet of Japanese quail boosts antioxidant capacity by elevating glutathione levels and catalase activity while decreasing malondialdehyde (50).

The reduction in malondialdehyde content signifies an enhancement in antioxidant defense and mitigation of oxidative stress, thereby safeguarding biological components from peroxidation and oxidation, as evidenced by prior research (69, 70). The enhancement of antioxidant capacity in the treated groups in the present study may result from probiotic bacteria utilizing their natural resources to neutralize extra free radicals and bolster the host's antioxidant capacity (71, 72).

The combination of *B. coagulans* and *P. polymyxa* enhanced antioxidant capacity and significantly (*P* < 0.05) improved immunological response in the supplemented groups. Probiotic dietary supplementation enhanced antibody production against sheep red blood cells and elevated antibody titers against Newcastle disease virus in meat-type quail (61). The outcomes indicated that administering a Bc+Pp in the quail diet enhanced globulin, serum immunoglobulin, and C3 levels. This aligns with Wu et al. (9), who showed that dietary *P. polymyxa* stimulates an immunological state by preserving equilibrium between pro-inflammatory and anti-inflammatory responses, thereby safeguarding intestinal homeostasis (9). Moreover, feeding *B. subtilis* increases IgM levels, with no observed variations in IgA and IgG levels in poultry breeders (73). Our results concur with those of Fathi et al. (20), who indicated that supplementing *B. subtilis* positively influences IgM levels in poultry subjected to heat stressors (20).

Additionally, probiotic administration enhanced humoral immunity by elevating serum IgA and IgM levels in poultry (74). Bai et al. (75) observed that the incorporation of probiotics derived from *B. subtilis* fmbJ into broiler feeds resulted in a significant (*P* < 0.05) rise in blood levels of both IgA and IgG. The elevated immunoglobulin levels in the treated groups may result from the immunomodulatory effects of *P. polymyxa* (24). The increased levels of IgM, IgA, and complement 3 in probiotic-supplemented groups suggest an enhancement of systemic humoral immunity in Japanese quails. IgM is typically associated with primary immune responses, while IgA plays a role in both mucosal and systemic immunity. The elevation in complement 3 further supports enhanced innate immune activity. The capacity of *P. polymyxa* to augment immunological state efficacy provides compelling justification for its application as an antibiotic alternative to improve animal well being and efficiency (20).

### Digestive enzymes and caecal microbiota

The prescription of probiotics improves growth performance by increasing the population of beneficial bacteria in the gut. The reduction in total coliform counts and the increase in lactic acid bacteria may indicate a shift toward a more balanced microbial profile in the cecum. According to Salah et al. (12), enhancing the function of the gut barrier promotes the production of digestive enzymes, which ultimately leads to an increase in the absorption of nutrients and helps to promote body weight and body weight gain, which aligns with our results.

Dietary supplementation with *P. polymyxa* 10 (BSC10) improved gut status by augmenting gut barrier function and enhancing the immunity of broilers (9). Dietary supplementation with *Paenibacillus xylanexedens*, as a probiotic, improved gut shape and decreased *E. coli* levels in the cecum of poultry (8). Prior research indicates that the administration of *Bacillus* spp. enhances gut microbial profile (19), promotes the colonization of beneficial bacteria in poultry (76), alters the caecal bacteria of broilers to a healthier equilibrium by providing helpful microorganisms and reducing potentially pathogenic microorganisms, and improves the interaction dynamics within the gastrointestinal microbiome (77).

The recent *in vitro* findings indicated that the isolated *P. polymyxa* LM31 has antibacterial properties through the production of antibiotics such as penicillin and polymyxin. Our findings indicated that the dietary mixture of *B. coagulans* and *P. polymyxa* elicited significant (*P* < 0.05) alterations in caecal microbiota, resulting in a decline in the amount of caecal *E. coli* and *Enterococcus* spp. with an increase in the count of caecal lactic acid bacteria. *P. polymyxa* has shown antibacterial properties against microorganisms using diffusible metabolites and bacterial volatiles as plant elicitors (78).

The inclusion of *B. subtilis* markedly decreased intestinal coliforms and *E. coli* in laying Japanese quails (79). The native *Lactobacillus* strains (150 g ton^−1^ food) included as probiotics markedly diminished *E. coli* proliferation, while *Lactobacillus* spp. proliferated in Japanese quail (62). Supplementation of *Bacillus* spp. reduced caecal *E. coli*, although caecal lactobacilli counts rose in poultry (80). Supplementation with *B. subtilis* C-3102 decreased caecal *E. coli* levels and elevated caecal lactobacilli counts in broiler chicks (81). Comprehensive research has been undertaken to clarify the pathogenic inhibitory effect of probiotics, yet the precise mechanism remains incompletely understood (2). *Bacillus* spp. may reduce infections through direct suppression, the synthesis of antimicrobial peptides, or by strengthening the mucosal layer of the intestine to obstruct microbial diffusion across the membrane (19).

Pathogenic management is a crucial concern for poultry producers and consumers, with significant economic and public health ramifications (19). The current findings demonstrated that a dietary combination of *B. coagulans* and *P. polymyxa* suppressed *E. coli* and *Enterococcus* spp. while concurrently promoting lactic acid bacteria. The suppression of detrimental bacteria may lead to a more resilient intestinal milieu, optimizing nutrient absorption, augmenting productivity, enhancing immunological function, and strengthening antioxidative capabilities.

## Conclusion

In summary, dietary supplementation with a probiotic combination of *B. coagulans and P. polymyxa* at a dosage of 1 mg kg^−1^ significantly (*P* < 0.05) enhanced growth performance, feed conversion efficiency, and various health-related metrics, encompassing liver and kidney function biomarkers, lipid profile, antioxidant capacity, immunological indicators, digestive enzyme activity, and caecal microbial load. The findings indicate that low-dose probiotic combinations may function as efficient natural performance enhancers for the growth of meat-type quail; nevertheless, more study is necessary to verify long-term safety and refine dosage methodologies.

## Data Availability

The original contributions presented in the study are included in the article/Supplementary material, further inquiries can be directed to the corresponding author.
