# Supplementary material for: Harnessing functional feed additives for sustainable production: the role of Bacillus coagulans and Paenibacillus polymyxa mixture in improving production and health of meat-type quails
Source: Front Vet Sci. 2025 Sep 24;12:1639681. doi: 10.3389/fvets.2025.1639681 (PMC12504081; doi:10.3389/fvets.2025.1639681)
Supplement: Supplementary file 1 [file Supplementary_file_1.docx]

***Supplementary Materials***

**Harnessing functional feed additives for sustainable production: the role of *Bacillus coagulans* and *Paenibacillus polymyxa* mixture in improving production and health of meat-type quails**

Fayiz M. Reda, Mahmoud Alagawany, Ayman S. Salah, Layla A. Almutairi, Mohammed A. Alqahtani, Soha A. Alamoudi, Saleh Altuwaijri, Khaled A. El-Tarabily*, Mohamed T. El-Saadony

*Correspondence: ktarabily@uaeu.ac.ae (Khaled A. El-Tarabily)

**TABLE S1** Screening *Bacillus* isolates (BcMT1–BcMT20) for antibacterial activity. Isolate BcMT15 was selected in the present study.

| Isolate | Inhibition zone (mm) (Mean ± SD) | | Selection status |
| --- | --- | --- | --- |
|  | *Staphylococcus aureus* | *Pseudomonas* *aeruginosa* |  |
| BcMT1 | 20.5 ± 0.7 | 18.2 ± 0.5 | - |
| BcMT2 | 22.1 ± 0.9 | 19.6 ± 0.6 | - |
| BcMT3 | 25.3 ± 1.2 | 22.0 ± 0.9 | - |
| BcMT4 | 23.7 ± 1.0 | 20.1 ± 0.8 | - |
| BcMT5 | 19.8 ± 0.6 | 16.5 ± 0.3 | - |
| BcMT6 | 24.2 ± 1.1 | 21.3 ± 0.7 | - |
| BcMT7 | 22.9 ± 0.8 | 19.8 ± 0.6 | - |
| BcMT8 | 21.0 ± 0.7 | 18.5 ± 0.5 | - |
| BcMT9 | 27.5 ± 1.4 | 24.3 ± 1.1 | Runner-up |
| BcMT10 | 21.3 ± 0.5 | 17.8 ± 0.4 | - |
| BcMT11 | 24.8 ± 1.0 | 21.5 ± 0.8 | - |
| BcMT12 | 22.4 ± 0.8 | 19.2 ± 0.6 | - |
| BcMT13 | 26.7 ± 1.3 | 23.1 ± 1.0 | Runner-up |
| BcMT14 | 23.1 ± 0.9 | 20.4 ± 0.7 | - |
| BcMT15 | 30.0 ± 1.5 | 26.0 ± 1.2 | Selected |
| BcMT16 | 25.8 ± 1.1 | 22.4 ± 0.9 | - |
| BcMT17 | 23.5 ± 1.0 | 20.7 ± 0.8 | - |
| BcMT18 | 21.9 ± 0.8 | 18.9 ± 0.6 | - |
| BcMT19 | 20.2 ± 0.7 | 17.3 ± 0.5 | - |
| BcMT20 | 22.6 ± 0.9 | 19.5 ± 0.7 | - |

**TABLE S2** Survival rate (%) of *Bacillus* isolates (BcMT3, BcMT9, BcMT13, and BcMT15) against low pH and bile salt (0.3%). Isolate BcMT15 was selected in the present study.

| Isolate | Inhibition zone (mm) (Mean ± SD) | | Survival rate (%) | |
| --- | --- | --- | --- | --- |
|  | *Staphylococcus aureus* | *Pseudomonas* *aeruginosa* | pH 2.5 | 0.3% bile salt |
| BcMT3 | 25.3 ± 0.2 | 22.0 ± 0.4 | 70.1 ± 3.0 | 65.4 ± 2.1 |
| BcMT9 | 27.5 ± 0.3 | 24.3 ± 0.2 | 79.4 ± 2.7 | 72.3 ± 2.5 |
| BcMT13 | 26.7 ± 0.5 | 23.1 ± 0.5 | 75.8 ± 2.9 | 68.9 ± 2.3 |
| BcMT15 | 30.0 ± 0.8 | 26.0 ± 0.3 | 85.2 ± 3.1 | 78.6 ± 2.8 |

**TABLE S3** Antibiotic resistance profiles of some selected *Bacillus* isolates (BcMT3, BcMT9, BcMT13, and BcMT15). Isolate BcMT15 was selected in the present study.

| Antibiotic (30 µg) | BcMT3 | BcMT9 | BcMT13 | BcMT15 | CLSI Interpretation |
| --- | --- | --- | --- | --- | --- |
| Tetracycline | 15 ± 0.7 | 18 ± 0.8 | 20 ± 0.9 | 22 ± 1.0 | S (≥19 mm) |
| Azithromycin | 19 ± 0.9 | 21 ± 1.0 | 23 ± 1.1 | 25 ± 1.2 | S (≥19 mm) |
| Erythromycin | 20 ± 1.0 | 23 ± 1.1 | 25 ± 1.2 | 28 ± 1.3 | S (≥19 mm) |
| Ceftriaxone | 12 ± 0.5 | 14 ± 0.6 | 16 ± 0.7 | 18 ± 0.8 | I (15–18 mm), R (≤14 mm) |
| Gentamicin | 22 ± 1.1 | 25 ± 1.2 | 28 ± 1.4 | 30 ± 1.5 | S (≥19 mm) |

Inhibition zone diameter, mm ± SD. Number of replicates = 3.

**TABLE S4** Hemolytic activity assessment of *Bacillus* isolates (BcMT3, BcMT9, BcMT13, and BcMT15). Isolate BcMT15 was selected in the present study.

| Isolate | Inhibition zone (mm) | Hemolysis type | Interpretation | Safety status |
| --- | --- | --- | --- | --- |
| BcMT3 | 1.2 ± 0.3 | α-hemolysis* | Partial hemolysis | Caution advised |
| BcMT9 | 0±0.0 | γ-hemolysis | Non-hemolytic | Safe |
| BcMT13 | 0±0.0 | γ-hemolysis | Non-hemolytic | Safe |
| BcMT15 | 0±0.0 | γ-hemolysis | Non-hemolytic | Safe |

**TABLE S5** Screening *Paenibacillus* isolates (PpMT1–PpMT55) for antibacterial activity. Isolate PpMT37 was selected in the present study.

| Isolate | Inhibition zone (mm) (Mean ± SD) | | Selection status |
| --- | --- | --- | --- |
|  | *Staphylococcus aureus* | *Pseudomonas* *aeruginosa* |  |
| PpMT1 | 18.2 ± 0.6 | 16.5 ± 0.5 | – |
| PpMT5 | 19.7 ± 0.8 | 17.3 ± 0.6 | – |
| PpMT8 | 25.2 ± 1.3 | 23.1 ± 1.1 | Runner-up |
| PpMT14 | 21.5 ± 0.9 | 19.2 ± 0.7 | – |
| PpMT19 | 22.3 ± 1.0 | 20.1 ± 0.8 | – |
| PpMT21 | 23.8 ± 1.1 | 21.4 ± 0.9 | – |
| PpMT29 | 26.7 ± 1.4 | 24.3 ± 1.2 | Runner-up |
| PpMT31 | 24.6 ± 1.2 | 22.0 ± 1.0 | – |
| PpMT33 | 20.1 ± 0.7 | 18.0 ± 0.5 | – |
| PpMT37 | 28.5 ± 1.6 | 26.8 ± 1.4 | Selected |
| PpMT41 | 24.1 ± 1.1 | 21.9 ± 0.9 | – |
| PpMT44 | 22.9 ± 1.0 | 20.7 ± 0.8 | – |
| PpMT46 | 21.0 ± 0.9 | 19.5 ± 0.7 | – |
| PpMT49 | 20.3 ± 0.8 | 18.2 ± 0.6 | – |
| PpMT55 | 19.8 ± 0.7 | 17.9 ± 0.5 | – |

**TABLE S6** Survival rate (%) of *Paenibacillus* isolates (PpMT8, PpMT29, PpMT31, and PpMT37) against low pH and bile salt (0.3%). Isolate PpMT37 was selected in the present study.

| Isolate | Inhibition zone (mm) | | | Survival rate (%) (Mean ± SD) | |
| --- | --- | --- | --- | --- | --- |
|  | *Staphylococcus aureus* | *Pseudomonas* *aeruginosa* | pH 2.5 | | 0.3% Bile salt |
| PpMT8 | 24.6 | 22.0 | 72.0 ± 2.3 | | 65.5 ± 2.1 |
| PpMT29 | 25.2 | 23.1 | 75.2 ± 2.5 | | 68.1 ± 2.3 |
| PpMT31 | 26.7 | 24.3 | 78.6 ± 2.6 | | 70.8 ± 2.4 |
| PpMT37 | 28.5 | 26.8 | 82.4 ± 2.9 | | 76.3 ± 2.7 |

**TABLE S7** Antibiotic resistance profiles of some selected *Paenibacillus* isolates (PpMT8, PpMT29, PpMT31, and PpMT37). Isolate PpMT37 was selected in the present study.

| Antibiotic (30 µg) | PpMT8 | PpMT29 | PpMT31 | PpMT37 | CLSI Interpretation |
| --- | --- | --- | --- | --- | --- |
| Tetracycline | 16 ± 0.7 | 17 ± 0.8 | 19 ± 0.9 | 21 ± 1.0 | S (≥19 mm) |
| Azithromycin | 18 ± 0.9 | 20 ± 1.0 | 22 ± 1.1 | 24 ± 1.2 | S (≥19 mm) |
| Erythromycin | 21 ± 1.0 | 22 ± 1.1 | 24 ± 1.2 | 27 ± 1.3 | S (≥19 mm) |
| Ceftriaxone | 14 ± 0.5 | 15 ± 0.6 | 17 ± 0.7 | 19 ± 0.8 | I (15–18 mm), R (≤14 mm) |
| Gentamicin | 24 ± 1.3 | 25 ± 1.3 | 28 ± 1.0 | 30 ± 1.2 | S (≥19 mm) |

Inhibition zone diameter, mm ± SD. Number of replicates = 3.

**TABLE S8** Hemolytic activity assessment of some selected *Paenibacillus* isolates (PpMT8, PpMT29, PpMT31, and PpMT37). Isolate PpMT37 was selected in the present study.

| Isolate | Hemolysis zone (mm) | Hemolysis type | Interpretation | Safety status |
| --- | --- | --- | --- | --- |
| PpMT8 | 1.0 ± 0.2 | α-hemolysis | Partial hemolysis | Caution advised |
| PpMT29 | 0 ± 0.0 | γ-hemolysis | Non-hemolytic | Safe |
| PpMT31 | 0 ± 0.0 | γ-hemolysis | Non-hemolytic | Safe |
| PpMT37 | 0 ± 0.0 | γ-hemolysis | Non-hemolytic | Safe |


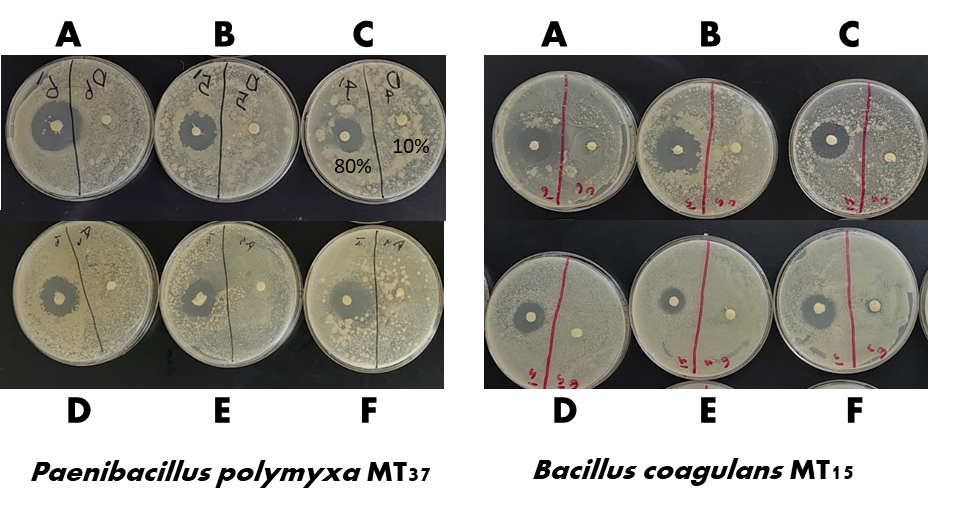


**Figure S1**

Antibacterial activity of *Bacillus coagulans* BcMT15 and *Paenibacillus polymyxa* PpMT37 against pathogenic Gram-positive (A, *Staphylococcus aureus*; B, *Streptococcus pyogenes*; and C, *Listeria monocytogenes*), and Gram-negative bacteria (D, *Salmonella typhi*; E, *Escherichia coli*; and F, *Klebsiella pneumoniae*).
